# A172 PSYCHOSOCIAL VARIABLES RELATED TO J-POUCH SURGERY FOR INFLAMMATORY BOWEL DISEASE: A SCOPING REVIEW

**DOI:** 10.1093/jcag/gwab049.171

**Published:** 2022-02-21

**Authors:** Q Hanna, D A Tripp, M J Beyak, P Moayyedi

**Affiliations:** 1 Psychology, Queen’s University, Kingston, ON, Canada; 2 Queen’s University, Kingston, ON, Canada; 4 McMaster University, Hamilton, ON, Canada

## Abstract

**Background:**

Ulcerative Colitis (UC) and Crohn’s Disease (CD) are subtypes of Inflammatory Bowel Disease (IBD) which is a condition with an unclear etiology causing inflammation of the small and large intestine (Public Health Agency of Canada, 2021). IBD is treated by diet, medications, and/or surgeries, with the most common surgery recommended to UC patients being j-pouch surgery (Crohn’s and Colitis Canada, n.d.). J-pouch surgery is often accompanied by numerous side effects (e.g., leakage, pouchitis), but it has been shown to help manage symptoms and restore function (Crohn’s and Colitis Canada, n.d.). While noteworthy research has examined the functional and biological outcomes of j-pouch surgery (e.g., Kayal et al., 2020), far less has considered the perioperative psychosocial implications.

**Aims:**

The aim of this scoping review is to assess the current literature concerning psychosocial factors related to j-pouch surgery for patients with IBD.

**Methods:**

We used the PRISMA-ScR (Tricco et al., 2018) and JBI recommendations (Peters et al., 2020) as methodological guidelines for conducting this review. We conducted our search during the summer and fall of 2021 and searched the following sources: *Medline, PsychInfo, CINAHL, EBM Reviews*, *ProQuest Dissertations and Theses Global, ResearchGate, Prospero,* and *PrePubMed*. We included articles from 1980-September 13, 2021.

**Results:**

Our initial search produced 718 articles. Of those that met our inclusion criteria, the majority discussed quality of life as their sole psychosocial variable. Among these studies, however, many used quality of life measures (e.g., IBDQ, SF-36) that prioritize health-related factors, with only a subset of questions directly addressing quality of life. The second most investigated psychosocial variables were those related to sexual health and functioning. When considering the levels of evidence, very few of our results were randomized control trials (RCT), while many were reviews (but not exclusively of RCT) and non-randomized controlled studies.

**Conclusions:**

We have identified a substantial need for studies examining psychosocial implications of and for j-pouch surgery among patients with IBD. In our discussion, we identify common variables and outline the strongest articles in the current literature, investigate the disadvantages of currently used measures, and propose specific directions for future research.

**Funding Agencies:**

None

